# *Teredinibacter turnerae* secretome highlights key enzymes for plant cell wall degradation

**DOI:** 10.1186/s40643-025-00876-7

**Published:** 2025-05-06

**Authors:** Lyle Ijssel P. De Guzman, Renato C. Carpina, Joan Catherine A. Chua, Eizadora T. Yu

**Affiliations:** 1 Institute of Chemistry, University of the Philippines Diliman, Quezon City, Philippines; 2 Marine Science Institute, University of the Philippines Diliman, Quezon City, Philippines

**Keywords:** *Teredinibacter turnerae*, Carbohydrate-active enzymes, Mass spectrometry, Biomass, Secretome

## Abstract

Carbohydrate-active enzymes (CAZymes) are crucial in the sustainable production of fuels and raw materials from recalcitrant plant cell wall polysaccharides (PCWPs). *Teredinibacter turnerae*, a symbiont of wood-boring shipworms, is a prolific degrader of plant biomass, largely due to the extensive CAZyme repertoire in its genome. To identify key enzymes involved in PCWP utilization, we analyzed the secretomes of *T. turnerae* E7MBN strain grown on sucrose, major PCWPs (cellulose, xylan, and pectin), and residual rice hull biomass using mass spectrometry-based proteomics. Our results show that *T. turnerae* E7MBN exhibits minimal enzyme secretion across various carbon sources, where secretomes mostly display similar functional profiles. Enzymatic complexity varied with the substrate, with cellulose-grown secretome being the most complex and comprising the majority of secreted CAZymes. These CAZymes contain domains that primarily target cellulose, hemicellulose, or pectin, notably including multicatalytic enzymes that are consistently found in the secretome and are likely central to biomass degradation. In contrast, the xylan-grown secretome displayed a more specific response, secreting only a single bifunctional hemicellulase, E7_MBN_00081, also identified as a core component of the bacteria’s enzymatic repertoire. Meanwhile, the pectin-grown secretome consists of multiple tonB-dependent receptors, which, along with isomerases, are considered common secretome constituents. E7MBN also demonstrated the capability to utilize rice hull biomass, predominantly secreting proteins previously identified under cellulose. Protein-protein interaction network analysis further revealed functional associations between CAZymes and several uncharacterized proteins, which include CBM-containing redox enzymes and a putative xylan-acting protein, thus offering new insights into their potential role in lignocellulose degradation. Overall, our work contributes to our understanding of enzymatic strategies employed by *T. turnerae* for PCWP deconstruction and highlights its potential as a promising source of CAZymes for sustainable biomass conversion.

**Graphical abstract:**

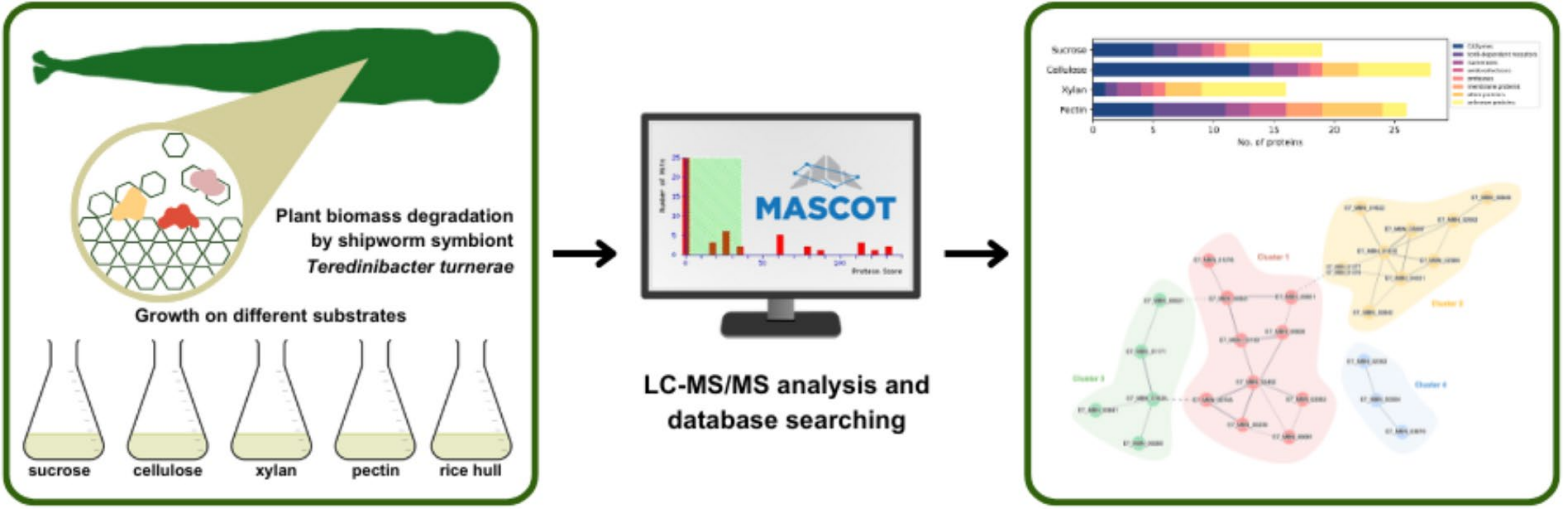

**Supplementary Information:**

The online version contains supplementary material available at 10.1186/s40643-025-00876-7.

## Introduction

The global climate crisis has driven a shift toward sustainable alternatives to petroleum-based products, with plant biomass as a promising resource. Central to this shift is the enzymatic degradation of plant cell wall-associated polysaccharides (PCWPs), including cellulose, hemicellulose, and pectin, which are all abundant carbon reservoirs (Bhardwaj et al. 2021). Carbohydrate-active enzymes (CAZymes) specifically target PCWPs to release fermentable sugars and intermediates, which can then be transformed into biofuel, bioplastics, and other value-added products (Østby et al. 2020). These proteins are crucial for developing strategies to harness PCWPs in various sectors, such as agriculture, textiles, and pharmaceuticals (Langsdorf et al. 2021; Lee et al. 2023; Sarangi et al. 2023; Shadhin et al. 2023).

The complex chemistry of plant polysaccharides requires multiple enzymes for efficient hydrolysis, prompting the use of enzyme mixtures in biorefineries (Adsul et al. 2020; Agrawal et al. 2018; Champreda et al. 2019; Malgas et al. 2017; Mohanram et al. 2013). Most commercially used enzymes are from fungi, such as the aerobic cellulolytic fungi *Trichoderma reesei*, known for its ability to secrete a wide range of CAZymes in large amounts (Borin et al. 2015; Jiang et al. 2022). However, challenges related to their growth, genetic intractability, and high enzyme production cost continue to limit the scalability of bioconversion processes (Bugg et al. 2011; Seppälä et al. 2017; Sethupathy et al. 2021). This highlights the need for alternative enzyme sources, such as bacteria from unique ecological niches, which may offer novel CAZymes with greater functional diversity and efficiency for biomass degradation.

The shipworm endosymbiont, *Teredinibacter turnerae*, is a promising source of lignocellulolytic enzymes. Isolated from the gill of wood-boring shipworms, commonly known in the Philippines as *tamilok*, *T. turnerae* is an intracellular aerobic gammaproteobacterium that specializes in degrading wood polysaccharides (Distel 2003; Distel et al. 2002). This endosymbiont aids its host in digesting wood by producing an array of lignocellulolytic enzymes that are secreted and translocated into the host’s cecum, where digestion primarily takes place (Giovanna Pesante et al.,^2021^; O’Connor et al. 2014; Sabbadin et al. 2018). Whole genome sequencing of *T. turnerae* T7901 strain reported an arsenal of CAZyme domains that predominantly encode for glycoside hydrolases (GHs) chiefly involved in degrading complex carbohydrates found in woody materials (i.e., cellulose, xylan, mannan, rhamnogalacturonan, and pectin) (Yang et al. 2009), suggesting a high degree of specialization towards plant biomass utilization. Furthermore, many of these enzymes contain an N-terminal signal peptide that allows extracellular secretion (Yang et al. 2009). Altogether, these suggest the abundance of CAZymes in the bacterial secretome from which new CAZymes can be discovered and sourced.

Shotgun proteomics has played a pivotal role in the characterization of microbial secretomes. Identification of key cellulose-degrading enzymes using MS-based proteomics has been reported for many cellulolytic bacteria, such *Ruminiclostridium papyrosolvens* (Ren et al. 2019), *Cellulomonas sp.* (Piccinni et al. 2019; Wakarchuk et al. 2016), and *Caldicellulosiruptor* (Lochner et al. 2011). In *T. turnerae*, MS-based profiling of the bacterial secretome grown in pure substrate is limited to a study by Yang et al. (2009), where they identified secreted proteins by the T7901 strain during growth in sucrose, Sigmacellulose (crystalline cellulose), and carboxymethylcellulose (CMC). However, only a portion of the secreted proteins was reported, and no further analysis was performed. On the other hand, a study by O’Connor et al. (2014) revealed that there is an abundance (98%) of endosymbiont-encoded proteins active against PCWPs detected in the cecum of the shipworm *Bankia setacea* (O’Connor et al. 2014). Secretion signal sequences were found in all endosymbiont-encoded genes detected in the cecum, suggesting that these proteins are selectively transported from their site of synthesis in the gills to the gut. CAZymes corresponding to endosymbiont-encoded proteins exhibited activity against cellulose and hemicellulose (e.g. mannan and xylan), consisting domains representative of GH families 5, 6, 9, 10, 11, 45, and 53, CE families 1, 3, 4, 6, and 15, and an enzyme with auxiliary activity (AA10) (O’Connor et al. 2014). Recently, it has been reported that *T. turnerae* strains T7901 and SR01903 secrete outer membrane vesicles that contain a diverse array of CAZymes with retained cellulolytic activity (Gasser et al. 2024). However, these findings are limited to growth in cellulose, raising interesting questions on the ability of *T. turnerae* to completely degrade plant cell walls, which include hemicellulose and pectin.

In this work, through a shotgun proteomics approach, we describe the profile of secreted proteins of a Philippine *T. turnerae* isolate during growth in different PCWPs and residual biomass. From this, we identified key proteins potentially involved in plant cell wall degradation and investigated previously unreported proteins that may contribute to the polysaccharolytic ability of *T. turnerae*. The results of this study reveal the enzymes and strategies employed by *T. turnerae* to degrade plant biomass, highlighting the potential of shipworm symbionts for applications in biofuel research and biotechnology.

## Methods

### Sampling and cultivation of local *T. turnerae* isolate

A local strain of *Teredinibacter turnerae*, E7MBN, was previously isolated from a bacterial consortium obtained from the gills of *Teredinidae sp.* sampled from the coastal areas of Mabini, Batangas, Philippines. E7MBN belongs to the same clade as the representative strain, T7901 (*NCBI-RefSeq: NC_012997*). E7MBN demonstrated stable growth in liquid media and significant cellulolytic activity during preliminary screening; hence, E7MBN was selected for secretome analysis. The collection of the host and the isolates complied with legal and regulatory provisions stated under Gratuitous Permit No. FBP-0036-10 and 0175-19 issued by the Department of Agriculture - Bureau of Fisheries and Aquatic Resources, Philippines (DA-BFAR).

E7MBN was cultivated in Shipworm Basal Medium (SBM) as previously described (Waterbury et al. 1983). SBM was supplemented with 0.2% (w/v) sucrose (Sigma-Aldrich, USA) as the sole carbon source and 0.03% (w/v) ammonium chloride. Liquid cultures were incubated at 30ºC with mild agitation (150 rpm) until turbid. After 2–3 days of growth, aliquots of the liquid culture, normalized to a final OD600 of ~ 0.100, were introduced to the growth medium containing either 0.2% (w/v) sucrose, crystalline cellulose (Sigmacell Cellulose Type 101, Sigma-Aldrich, USA), xylan (Xylan from beechwood, Sigma-Aldrich, USA), or pectin (Pectin from citrus peel, Sigma-Aldrich, USA) as the sole carbon source. All cultivations were done in triplicate.

Rice hull was also used as a growth substrate to determine whether E7MBN can degrade plant biomass. The rice hull was ground in a blender until pulverized and passed through an ultrafine sieve (250 μm) before sterilizing in an autoclave. The inoculum was introduced to SBM supplemented with 0.2% (w/v) rice hull suspension. Cultivation in rice hull suspension was performed for two biological replicates.

### Enzymatic activity measurement

Enzymatic activity of crude supernatants against PCWPs was measured via DNS assay. Activity assays of the secreted proteins were performed by incubating the culture supernatants in a reaction mixture containing 0.5% (w/v) substrates: carboxymethyl cellulose (CMC, Sigma-Aldrich, USA), beechwood xylan (Sigma-Aldrich, USA), pectin from citrus peels (Sigma-Aldrich, USA) in 50 mM HEPES buffer pH 8.0 at 30ºC for 1 h. The concentration of reducing sugars released was measured by the dinitrosalicylic acid (DNS) method at 540 nm (Miller 1959). One unit activity (U) is defined as the amount of enzyme required to release 1 µmol reducing sugar per minute (1 U = 1 µmol min^− 1^). All measurements were performed in triplicate.

### Protein isolation and SDS-PAGE

Bacterial cultures were harvested after two (2) days of growth and cleared of cells by centrifugation and filtration through a 0.22 μm syringe filter. Secretome proteins were concentrated using a three (3) kDa molecular weight cut-off (MWCO) Pierce Protein Concentrator with PES membrane (ThermoFisher). Total protein concentration was determined via BCA assay. Protein concentrates (~ 10 µg) were treated with 2X Laemmli buffer (Sigma-Aldrich) and boiled for 5 min before loading in a 12% polyacrylamide gel (FastCast Acrylamide Kit, Bio-Rad). Proteins were visualized through Coomassie staining.

### Reduction-alkylation and in-solution trypsin digestion

Proteins were precipitated using chilled acetone and were resuspended in 50 mM ammonium bicarbonate pH 8.0 to a final concentration of ~ 1 mg/mL. Approximately 100 µg of protein was reduced with 55 mM dithiothreitol (DTT, Vivantis) at 60ºC for 30 min and alkylated with 55 mM iodoacetamide (IAM, Sigma-Aldrich) at 37ºC in the dark for 1 h. Proteins were then precipitated with chilled acetone, washed to remove excess DTT and IAM, and immediately resuspended in 50 mM ammonium bicarbonate, pH 8.0 to produce a ~ 1 mg/mL solution. In-solution trypsin (Promega) digestion was performed in 1:40 trypsin: protein ratio at 37ºC overnight. The reaction was stopped by adding acetonitrile with 1% formic acid. Tryptic digests were then transferred into an ANSI polypropylene 350-µL plate for MS analysis.

### Liquid chromatography and mass spectrometry analysis

Proteomic analysis of the E7MBN secretome was performed using an Acquity Ultra Performance Liquid Chromatography (UPLC) H-Class system with an electrospray ionization (ESI) quadrupole time-of-flight (qTOF) mass spectrometer (Xevo G2-S2, Waters). For PCWP-grown secretomes, samples were injected at 10 µL injection volume (~ 10 µg) into a Phenomenex Kinetex C18 UHPLC column (50 × 2.1 mm, 1.7 μm) maintained at 30.0ºC. Chromatographic separation was performed at a flow rate of 0.3 mL/min using solvent A (water + 0.1% formic acid) and solvent B (acetonitrile + 0.1% formic acid). Peptides were eluted using an 8-minute concentration gradient: 6.5 min gradient from 5 to 40% B, followed by 1.5 min gradient from 40 to 95% B. For proteins from sucrose cultures, peptide samples were injected and separated in a Waters CORTECS T3 column (100 × 2.1 mm, 1.6 μm) using the same flow rate and solvent system. Peptide elution was facilitated by a 15-minute concentration gradient: 12 min gradient from 5 to 40% B, followed by 3 min gradient from 40 to 95% B.

MS data acquisition was performed in positive mode using the following parameters: 2.80 kV capillary voltage, 40.00 V cone voltage, 80.00 V offset potential, and source and desolvation temperatures of 120ºC and 450ºC, respectively. MS1 masses were scanned within the 400–2000 m/z mass range for 0.5 s. Fast data-dependent acquisition (DDA) using the same scan time and a precursor window of 50–2000 m/z was performed to generate MS/MS spectra of precursor ions with intensity greater than 1.0 × 10^5^ ion counts and charge states of + 2 to + 4. Collision-induced dissociation (CID) using argon was carried out using collision energies of 20–30 V for low masses and 40–60 V for high masses. MS/MS data for the eight most abundant product ions were acquired per scan time.

### Protein identification

MS/MS raw data were processed using MASCOT ver. 2.7.0 (Matrix Science) software. Peak lists were generated using default parameters and subjected to MS/MS ion search using MASCOT. The annotated proteome of E7MBN was used as a custom database. The following parameters were set for the database search: fixed modification of carbamidomethylation of cysteine residues; variable modifications of methionine and proline oxidation, carboxylation of glutamic acid, and C-term amidation; maximum of 2 missed cleavages for trypsin; peptide mass tolerance of 10 ppm; and fragment ion mass tolerance of 0.05 Da. A decoy search was simultaneously performed, and the false discovery rate for peptide spectrum matches above homology was adjusted to 1%. Representative protein sequences from each protein family were compiled to generate a master list of unique protein groups. MASCOT automatically assigns a representative protein based on MOWSE scores, the number of peptide spectral matches, and protein coverage. Proteins with at least one (1) unique peptide match (with a minimum peptide length of 6 amino acids) in two of the three biological replicates were considered for identification.

Putative CAZymes in the final list were annotated using HMMER, dbCAN-sub, and DIAMOND databases via dbCAN3 server (https://bcb.unl.edu/dbCAN2/*)* (Zheng et al. 2023). Protein classifications and molecular functions were inferred using InterPro (https://www.ebi.ac.uk/interpro/*)* (Paysan-Lafosse et al. 2023), and Cluster of Orthologous Genes (COG, https://www.ncbi.nlm.nih.gov/research/cog) annotations (Galperin et al. 2015) annotations Localization and secretion mechanism were predicted using SignalPv6.0 (https://services.healthtech.dtu.dk/services/SignalP-6.0/*)* (Teufel et al. 2022) and SecretomeP 2.0 (https://services.healthtech.dtu.dk/services/SecretomeP-2.0/*)* (Bendtsen et al. 2005). Functional and physical protein-protein interactions (PPI) were analyzed using the STRING v.12 network database (https://string-db.org/; minimum required interaction score: medium confidence 0.400), and the MCL clustering (inflation parameter = 1.5) was used (Szklarczyk et al. 2023).

### Phylogenetic and structural analysis of E7_MBN_00801

The putative structure of E7_MBN_00801 was predicted using AlphaFold 3 (Abramson et al, 2024), showing two distinct domains, referred to as domA and domB. To infer protein identity and function, sequence alignment and phylogenetic analysis were performed for each domain. Similar sequences were obtained by searching the protein sequences against the NCBI nr database using the PSI-BLAST tool. The top 100 sequences were obtained after five (5) iterations and were used to construct a neighbor-joining tree via Molecular Evolutionary Genetics Analysis (MEGA) software (Version 11.0.13). Bootstrap values were obtained after 100 iterations. The resulting phylogenetic trees were pruned, leaving only the sequences that are closely related to the query protein.

## Results

### Enzymatic activity of crude secretomes

The *T. turnerae* E7MBN strain was cultured in minimal media (sucrose, E7S) and growth media supplemented with model PCWPs (cellulose, E7C; xylan, E7X; pectin, E7P). Enzymatic activity of each of the enzyme supernatants was tested against multiple substrates, including CMC, xylan, and pectin, to assess the specificity of the secreted enzyme mixtures (Table 1). As expected, each PCWP-grown secretome was active against the same carbon source on which the bacterium was cultivated, with only E7P capable of degrading pectin. All secretomes acted on more than one substrate, except for E7X which displayed high specificity for xylan only. Proteins from the E7C secretome demonstrated activity against CMC and xylan. Interestingly, all secretomes showed activity for and were most active against xylan, suggesting enhanced effectiveness for xylan deconstruction. This was significantly observed for E7X, which demonstrated the highest xylanolytic activity among all growth conditions. Visualization of the secreted proteins in a 12% polyacrylamide gel shows a slight variation in the band profile across the four secretomes (Figure S1). Majority of the detected proteins have molecular weights ranging from 45 to 240 kDa, some of which are consistently observed in all culture conditions.

**Table 1:** Enzymatic activity of crude secretomes in sucrose (E7S), cellulose (E7C), xylan (E7X), and pectin (E7P) against various plant polysaccharides

	Activity (U/mg)		
	Xylan	CMC	Pectin
**E7S**	1.94 ± 0.24	0.65 ± 0.32	ND
**E7C**	7.04 ± 1.36	1.43 ± 0.26	ND
**E7X**	11.3 ± 1.1	ND	ND
**E7P**	4.16 ± 1.12	0.60 ± 0.14	0.44 ± 0.096

*ND - not detected

### Proteomic analysis of E7MBN secretome in minimal media and PCWPs

#### Secretome profiles of E7MBN cultured in sucrose, cellulose, xylan, and pectin media

**Fig. 1:**
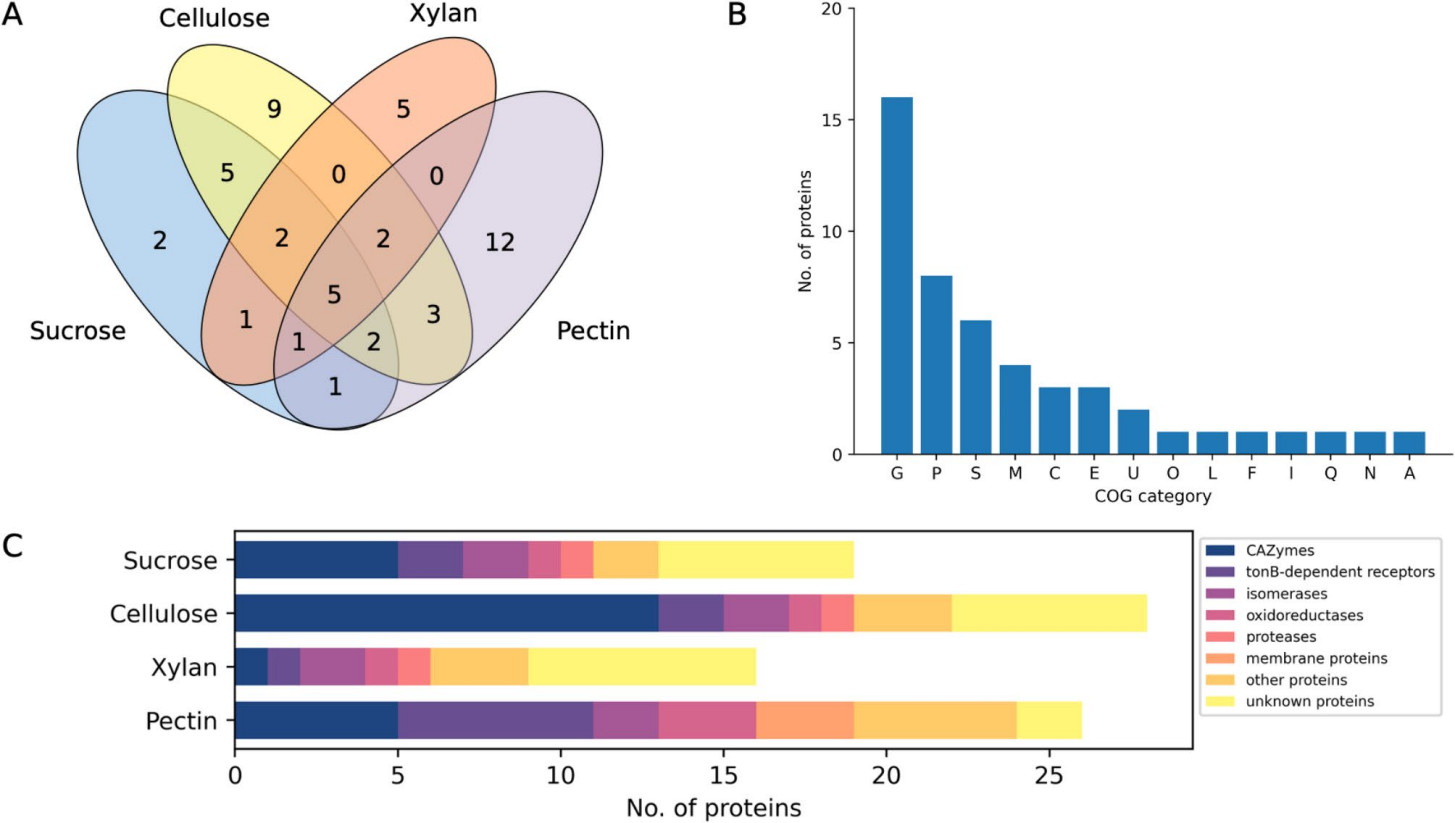
Distribution and functional classification of proteins identified in the E7MBN secretomes. (**A**) Venn diagram representing the number of unique and common proteins reported in E7MBN cultures grown under sucrose, cellulose, xylan, and pectin. (**B**) Functional profile of the secretome proteins according to COG annotation (G, carbohydrate transport and metabolism; P, inorganic ion transport and metabolism; S, function unknown; M, cell wall/membrane/envelope biogenesis; C, energy production and conversion; E, amino acid transport and metabolism; U, intracellular trafficking; O, post-translational modification, protein turnover, chaperones; L, replication, recombination, and repair; F, nucleotide transport and metabolism; I, lipid transport and metabolism; Q, secondary metabolites biosynthesis, transport, and catabolism; N, cell motility; A, RNA processing and modification). (**C**) Functional classification of secreted proteins by E7MBN

Total extracellular proteins secreted under minimal media and three model PCWPs were collected and analyzed by LC-MS/MS. Proteins with at least one (1) unique peptide match detected in two of the three biological replicates were considered. Fifty (50) proteins were identified across all culture supernatants, among which 19 were found in E7S, 28 in E7C, 16 in E7X, and 26 in E7P (Table 2). Most proteins (56%) were detected exclusively in secretomes obtained from a single carbon source, 17 proteins were present in secretomes obtained from at least two carbon sources and five proteins were detected in all cultures (Fig. 1A; Table 2). Thirty-two (32) proteins were reported to contain signal peptide sequences, six proteins were predicted to be secreted through non-classical pathways, and the remaining proteins could either be intracellular or have unknown localization.

**Table 2:** Proteins identified in culture supernatants of *T. turnerae* E7MBN grown on sucrose (S), cellulose (C), xylan (X), and pectin (P) by LC-MS/MS. T7901 homologs were identified using the UniProt database and signal peptides were predicted via SignalPv6.0. Non-classical secretion mechanism was assigned to proteins lacking signal peptides and scoring above 0.5, as predicted by secretomep 2.0 server

Accession code	Annotation	T7901 UniProt accession	Mass (Da)	Signal peptide	Predicted secretion mechanism	Growth substrate
E7_MBN_00021	hypothetical protein	C5BRG0	70,405	N	Non-classical	C
E7_MBN_00081	endoxylanase/acetylxylan esterase	C5BRL9	100,896	Y	Classical	S, C, X, P
E7_MBN_00085	5’-nucleotidase	C5BS06	73,091	Y	Classical	X
E7_MBN_00086	Endonuclease	C5BS07	77,867	Y	Classical	S, C
E7_MBN_00239	endoxylanase/acetylxylan esterase	C5BQU7	126,570	Y	Classical	C, P
E7_MBN_00260	carbohydrate binding protein	C5BSV8	198,086	Y	Classical	S, C
E7_MBN_00368	Outer membrane efflux protein	C5BTK9	33,788	Y	Classical	P
E7_MBN_00451	uncharacterized protein	C5BTU2	62,302	N	Non-classical	C
E7_MBN_00452	putative lipoprotein	C5BTU3	45,401	N	-	S
E7_MBN_00497	unknown	K7FWY9	22,618	N	-	S, X, P
E7_MBN_00624	Uncharacterized protein conserved in bacteria (DUF2170)	C5BI44	14,917	N	-	C, X, P
E7_MBN_00645	AAA ATPase containing von Willebrand factor type A	C5BKR9	145,405	Y	Classical	S
E7_MBN_00745	endoglucanase	C5BIG0	60,354	Y	Classical	C
E7_MBN_00801	putative GH8 endoxylanase	C5BMW2	31,539	N	Non-classical	X
E7_MBN_00820	glucanase/endoxylanase	C5BMU2	97,022	Y	Classical	S, C
E7_MBN_00821	cellodextrinase	C5BMU1	75,522	Y	Classical	C
E7_MBN_01082	IgGFc binding protein	C5BLC8	67,114	Y	Classical	C
E7_MBN_01104	Trypsin-like serine protease	C5BKX1	46,961	Y	Classical	S, C, X
E7_MBN_01171	Lytic polysaccharide monooxygenase	C5BKQ9	35,628	Y	Classical	C
E7_MBN_01276	Belongs to the xylose isomerase family	C5BKF1	49,800	N	-	S, C, X, P
E7_MBN_01277	TonB-dependent Receptor Plug Domain	C5BKF0	125,860	Y	Classical	S, C, P
E7_MBN_01278	carbohydrate binding domain-containing protein	C5BKF0	38,393	Y	Classical	S, C, P
E7_MBN_01415	Phosphopantetheine attachment site	C5BU60	8840	N	-	S, P
E7_MBN_01610	TonB-dependent Receptor Plug Domain	C5BT80	106,795	Y	Classical	P
E7_MBN_01622	TonB dependent receptor	C5BT68	98,542	Y	Classical	P
E7_MBN_01628	Amb_all (PL1_11(95–273) + PL1_11(663–777) + PL1_11(970–1153))	C5BT62	133,563	Y	Classical	P
E7_MBN_01784	Acetohydroxy acid isomeroreductase, catalytic domain	C5BQZ6	36,886	N	-	P
E7_MBN_02062	Alpha-L-arabinofuranosidase C-terminus	C5BPF6	57,636	Y	Classical	C
E7_MBN_02305	Pyridine nucleotide-disulphide oxidoreductase, dimerisation domain	C5BL84	51,732	N	-	C, X, P
E7_MBN_02330	hypothetical protein	C5BLP2	17,576	Y	Classical	S, C
E7_MBN_02352	Triosephosphate isomerase	C5BPZ9	26,354	N	-	S, C, X, P
E7_MBN_02452	Polysaccharide lyase	C5BQL8	97,043	Y	Classical	C
E7_MBN_02455	Bacterial protein of unknown function (DUF839)	C5BQM1	66,777	Y	Classical	S, X
E7_MBN_02489	Metallo-beta-lactamase superfamily	C5BQQ2	31,539	Y	Classical	C, P
E7_MBN_02518	OmpA family	C5BQS8	18,105	Y	Classical	P
E7_MBN_02552	hypothetical protein	C5BQQ5	97,319	Y	Classical	S, C, X
E7_MBN_02842	TonB dependent receptor	C5BN15	111,955	Y	Classical	S, C, X, P
E7_MBN_02908	Inorganic pyrophosphatase	C5BNL4	20,253	N	-	P
E7_MBN_02984	TonB dependent receptor	C5BNU1	106,591	Y	Classical	P
E7_MBN_03147	Bacterial flagellin C-terminal helical region	C5BRS9	59,841	N	Non-classical	P
E7_MBN_03193	Belongs to the glycosyl hydrolase family 6	C5BNB1	40,736	N	Non-classical	C, P
E7_MBN_03304	Iron/manganese superoxide dismutases, alpha-hairpin domain	C5BP07	21,574	N	Non-classical	S, C, X, P
E7_MBN_03347	Semialdehyde dehydrogenase, NAD binding domain	C5BP49	37,813	N	-	P
E7_MBN_03459	Esterase-like activity of phytase	C5BMI1	90,559	Y	Classical	X
E7_MBN_03697	TonB dependent receptor	C5BK74	114,053	Y	Classical	P
E7_MBN_03841	carbohydrate binding protein	C5BJQ6	85,244	Y	Classical	C
E7_MBN_03865	Glucose / Sorbosone dehydrogenase	C5BLQ7	143,269	Y	Classical	S, C

Proteins were classified according to the categories defined by the Cluster of Orthologous Groups (COG) to describe the functional associations within the secretome (Fig. 1B). A variety of COGs was identified, with majority of proteins enriched in categories G (carbohydrate transport and metabolism) and P (inorganic ion transport and metabolism), and some annotated as S (function unknown) and M (cell wall/membrane/envelope biogenesis). The highest number of proteins was consistently reported for COG category G for each growth condition, suggesting that E7MBN mainly secretes proteins related to carbohydrate processes during growth in liquid culture.

The secretomes encompass various protein groups, including CAZyme domain-containing proteins, membrane proteins, isomerases, and unknown proteins. Notably, 14 CAZyme domain-containing proteins were identified across all secretomes, representing 28% of the total proteins. This group includes glycoside hydrolases (GH), carbohydrate esterases (CE), polysaccharide lyases (PL), carbohydrate-binding proteins, and auxiliary enzymes (AA). Cell membrane-associated proteins, such as tonB-dependent receptors (TBDRs), transporters, and outer membrane proteins, comprise 18% of the secretome. Non-CAZy enzymes were also present, including isomerases, oxidoreductases, and proteases. Other known proteins (i.e., proteins involved in nucleotide metabolism and phosphatases) comprised 11% of the secretome, while unknown proteins accounted for the rest (23%).

A detailed analysis of each growth condition revealed distinct patterns in the distribution of proteins across different carbon sources (Fig. 1C). CAZymes and unknown proteins constitute a substantial portion of E7S and E7C secretomes, while E7X is largely comprised of unknown proteins. TBDRs, along with CAZymes and other proteins represented most of the proteins secreted in E7P. Comparing the proteomic profile between secretomes under minimal media and PCWPs, E7C appears to be highly similar with E7S, sharing almost two-thirds of proteins secreted when grown in sucrose. Notably, E7MBN secreted the most CAZymes (13 proteins) during growth in cellulose, comprising nearly half of the proteins identified in E7C. This contrasts with E7X and E7P, where CAZymes accounted for less than a fifth of the total identified proteins. In fact, only a single CAZyme was detected in E7X. Interestingly, CAZymes identified in E7C encompass almost all CAZymes found in the four growth conditions, with only one unique CAZyme reported in E7P (Table 3). The relatively low diversity of CAZymes across secretomes suggests minimal secretion in E7MBN, where a limited range of enzymes are utilized under various conditions. Meanwhile, CAZyme complexity appears to be selective and targeted to the carbon source, with E7C exhibiting the highest enzyme complexity and E7X comprising only a single CAZyme.

**Fig. 2:**
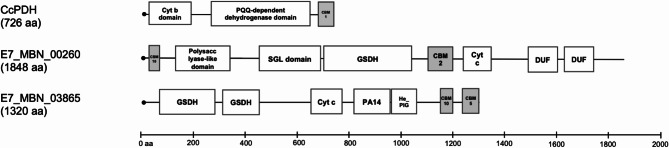
Domain architecture of the novel AA12 redox enzyme CcPDH from *Coprinopsis cinerea* and CBM-containing redox proteins found in the *T. turnerae* E7MBN secretome

In addition to CAZymes, proteins implicated in carbohydrate utilization, such as TBDRs and isomerases were consistently identified in the E7MBN secretomes. Two isomerases (xylose isomerase, E7_MBN_01276; triosephosphate isomerase, E7_MBN_02352) and one TBDR (E7_MBN_02842) were present in all carbon sources. Most TBDRs were exclusively detected in E7P, suggesting that secretion of TBDRs may be closely linked to pectin utilization.

#### Plant cell wall-degrading proteins in the E7MBN secretomes

Effective deconstruction of the plant cell wall depends on the cooperative action of CAZymes, which are a specialized group of enzymes that act on glycosidic linkages and other bonds found in complex carbohydrates. The E7MBN secretome consisted of 14 CAZymes across four growth conditions, most of which contain catalytic modules associated with degrading cellulose and hemicellulose (Table 3). The most represented CAZymes in the secretome are active against hemicellulose and typically contain domains with putative ​​endo-β -1,4-xylanase (GH10 and GH11) and acetylxylan esterase (CE1 and CE6) activities. Interestingly, the bifunctional hemicellulase, E7_MBN_00081, was consistently expressed and secreted in all substrates. Identified hemicellulases also include an ⍺-arabinofuranosidase (GH51), which was found in E7C. Cellulose-degrading enzymes were also present, occurring mostly in the cellulose-grown secretome. These include the exo-acting cellobiohydrolase (GH6) and members of the GH5 family, which are annotated as endoglucanase and cellodextrinase. Proteins that can act on pectin linkages were only secreted under cellulose and pectin, with rhamnogalacturonan lyase (PL11) detected in E7C and pectate lyase (PL1) in E7P. Most of the CAZymes are associated with carbohydrate-binding modules (CBMs), which allow binding to specific substrates, such as cellulose (CBM10) and xylan (CBM60), or to multiple polysaccharides (CBM2, 4, 5, and 35).

The secretome also consisted of other proteins related to carbohydrate degradation, including the AA10 lytic polysaccharide monooxygenase (LPMO). This protein was solely secreted under cellulose and is homologous to the previously characterized AA10 LPMO from *T. turnerae* T7901 (InterPro: C5BKQ9; PDB: 6RW7) (Fowler et al. 2019). Two proteins containing CBMs associated with non-CAZyme domains (E7_MBN_00260 and E7_MBN_03865) were also identified and are exclusively shared between E7C and E7S.

**Table 3:** Carbohydrate-active enzymes and accessory proteins involved in carbohydrate utilization identified in E7MBN secretomes grown on sucrose (S), cellulose (C), xylan (X), and pectin (P) by LC-MS/MS. CAZyme modules were predicted using dbCAN3

Protein ID	Predicted function	Domain Architecture	Secreted?	Substrate
E7_MBN_00081	endoxylanase/acetylxylan esterase	CE6 + CBM5 + CBM10 + CBM60 + GH10	Y	S, C, X, P
E7_MBN_00239	endoxylanase/acetylxylan esterase	GH11 + CBM60 + CBM5 + CBM57 + CE1 + CE1	Y	C, P
E7_MBN_00260	carbohydrate binding protein	CBM10 + CBM2	Y	S, C
E7_MBN_00745	endoglucanase	GH5 + CBM10 + CBM10 + CBM10	Y	C
E7_MBN_00820	glucanase/endoxylanase	GH11 + CBM5 + CBM10 + GH5	Y	S, C
E7_MBN_00821	cellodextrinase	CBM5 + CBM10 + GH5	Y	C
E7_MBN_01171	lytic polysaccharide monooxygenase	AA10 + CBM10	Y	C
E7_MBN_01278	carbohydrate binding protein	CBM4 + CBM4	Y	S, C, P
E7_MBN_01628	pectate lyase	PL1 + CBM5 + CBM35 + PL1	Y	P
E7_MBN_02062	⍺-arabinofuranosidase	GH51	Y	C
E7_MBN_02452	polysaccharide lyase	PL11 + CBM35 + CBM2	Y	C
E7_MBN_03193	cellobiohydrolase	GH6	N	C, P
E7_MBN_03841	carbohydrate binding protein	CBM2	Y	C
E7_MBN_03865	glucose/sorbosone dehydrogenase	CBM5 + CBM10	Y	S, C

#### Dominance of multi-catalytic enzymes in the E7MBN secretome

A notable feature of the E7MBN secretome is the presence of multicatalytic enzymes across all carbon sources. Unlike typical multidomain CAZymes that comprise a single catalytic domain with several CBMs, these enzymes consist of multiple catalytic domains with discrete activities. We identified four such CAZymes, which include three bifunctional hemicellulases and one pectate lyase. Two bifunctional hemicellulases (E7_MBN_00081, E7_MBN_00239) possess ​​endo-β -1,4-xylanase (GH10, GH11) and acetylxylan esterase (CE1, CE6) domains, while one (E7_MBN_00820) displays domains with putative endo-β -1,4-xylanase (GH11) and glucanase (GH5) functionalities. Meanwhile, the pectate lyase, E7_MBN_01628, is notable for its two PL1 domains. Multicatalytic CAZymes identified in the secretome feature domain combinations homologous to those found in the T7901 strain, except for E7_MBN_00239 which consists of a GH11 endoxylanase with two CE1 domains. BLAST analysis showed that the protein is most identical to TERTU_3447 from T7901 strain, which contains GH11 and CE15 domains instead (66.62% identity, 50% coverage). Alignment of the predicted protein structures revealed that the CE15 domains in TERTU_3447 are distinct from the two CE1 domains of E7_MBN_00239 (data not shown), suggesting that E7_MBN_00239 may be unique and thus, is an exciting subject for further investigation.

While only a limited number of multicatalytic CAZymes have been characterized to date, it has been shown that these enzymes could facilitate the synergistic degradation of complex carbohydrates (Chu et al. 2019; Kmezik et al. 2021; Krska et al. 2021). This could benefit E7MBN as secretion of such enzymes could enable efficient breakdown of the plant cell wall with reduced enzymatic repertoire. Correspondingly, multicatalytic enzymes may play a central role in the E7MBN secretome, suggested by the fact that the bifunctional endoxylanase/acetylxylan esterase, E7_MBN_00081, was the sole CAZyme identified in all carbon sources and the only one detected in E7X. Homologs of this protein have been identified in the Sigmacell-fed secretome of *T. turnerae* T7901 (Yang et al. 2009) and among the prokaryotic CAZymes found in the cecum of the shipworm *Bankia setacea* (O’Connor et al. 2014), suggesting its critical role in polysaccharide degradation within the shipworm symbiotic community.

#### CBM-containing redox proteins in cellulose-grown culture

Auxiliary proteins aid in PCWP utilization through synergistic action with hydrolytic enzymes. We found only one such protein, AA10 LPMO (E7_MBN_01171), in the secretome. However, several proteins with both CBM- and redox-active domains were also identified. These proteins, E7_MBN_00260 and E7_MBN_03865, are homologous to T7901 proteins TERTU_3803 (99.8% identity) and TERTU_2567 (98.9% identity), respectively, which have been found to contain pyrroloquinoline-quinone (PQQ)-dependent glucose/sorbosone dehydrogenase (GSDH) and cytochrome c (Cyt c) domains in their sequence (Fig. 2). Curiously, the combination of such domains appears to resemble the novel AA12 redox enzyme (CcPDH) from *Coprinopsis cinerea*. This protein, which also consists of cytochrome and PQQ-dependent dehydrogenase domains along with a CBM domain, has been reported to bind insoluble cellulose and drive LPMO activation (Takeda et al. 2015; Várnai et al. 2018). However, it remains to be seen whether the *T. turnerae* GSDH and Cyt c domains could function similarly, considering that while the *T. turnerae* proteins and CcPDH share common motifs, they display little sequence similarity (Fig. 2).

Heme-containing redox proteins typically work synergistically with other LPMOs through coupled electron transfer systems (Kracher et al. 2016). However, a recent study by Rajagopal et al. (2024) points to a potentially different mechanism. Structural investigations on the Cyt c and GSDH domains of the T7901 redox protein, TERTU_3803, revealed that these domains are incapable of donating electrons to LPMO (Rajagopal et al. 2024). As suggested by Rajagopal et al., it is therefore possible that these *T. turnerae* proteins participate in redox processes aside from LPMO biochemistry to contribute to cellulose degradation in the shipworm. 

### Protein-protein interaction (PPI) analysis of the total E7MBN secretome profile

**Fig. 3:**
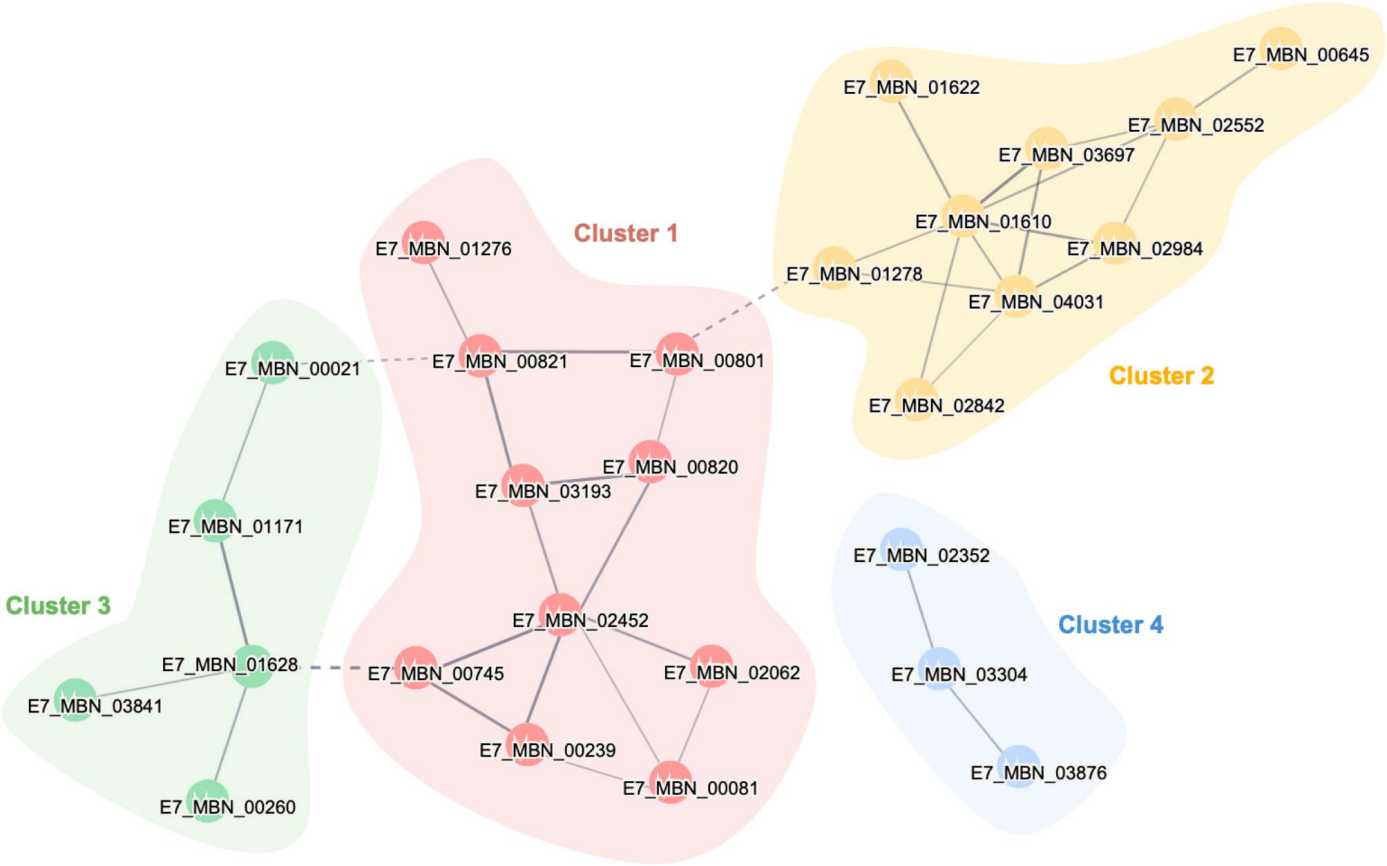
Protein-protein interaction (PPI) analysis via STRING (PPI enrichment value = 1.48e-7; medium confidence). The network consists of a total of 47 nodes, representing the proteins, with 50 edges indicative of interaction evidence. Intercluster relationships are represented as dashed lines. Proteins detected in the model substrates formed four main clusters: Cluster 1, carbohydrate metabolism (p-value = < 1e-16; red nodes); Cluster 2, cellular transport (p-value < 1.0e-16; orange nodes); and Cluster 3, CBD II domain, (p-value = 7.26e-06; green nodes), Cluster 4 (p-value = 2.08e-04; blue nodes)

We conducted a PPI network and enrichment analysis via STRING (Szklarczyk et al. 2023) to describe and gain insights on the functional associations among proteins identified in the E7MBN secretome. Proteins were searched against the T7901 proteome and annotated based on sequence homology to generate the PPI network (Fig. 3). A total of 47 nodes and 40 edges comprise the protein network. Some nodes are represented by more than one protein, such as the TBDR (E7_MBN_01277) and carbohydrate-binding protein (E7_MBN_01278), both of which displayed high similarity to the T7901 tonB-dependent receptor TERTU_4665 (only shown in the network as E7_MBN_01278). Meanwhile, proteins without homologs in T7901 were excluded from the network. The high number of predicted interactions suggests significant associations among the secretome proteins, possibly working toward a common biological function. However, not all proteins formed substantial networks, and a few were only displayed as disconnected nodes (not shown in Fig. 3).

Proteins were grouped into clusters corresponding to their biological functions and relationships. Four main protein clusters were obtained, three of which comprised most of the network, while some proteins formed smaller clusters (clusters with ≤ 2 nodes not shown). The biggest cluster (Cluster 1) consists of 10 proteins (p-value = 1.0e-12; red nodes) and is enriched in carbohydrate metabolic processes. CAZymes dominated this cluster, along with a xylose isomerase (E7_MBN_01276) and an unknown protein (E7_MBN_00801). Interactions in this cluster are not exclusively linear, and extensive connections between CAZymes could be observed with some proteins at a converging point in the network, reflecting a possible key role in polysaccharide degradation. For example, the polysaccharide lyase (E7_MBN_02452) interacts with six other CAZymes and serves as a hub among the proteins, possibly mediating various roles within the secretome.

The xylose isomerase E7_MBN_01276 was commonly identified in all carbon sources and has also been implicated as a core *T. turnerae* secretome protein (Yang et al. 2009). While this protein was predicted to be cytoplasmic, several xylose isomerases have also been identified in the metasecretomes of thermophilic and lignocellulolytic microbial consortia (D’haeseleer et al. 2013; Jiménez et al. 2015). Xylose isomerase enables the metabolic conversion of monosaccharides derived from the degradation of PCWPs, which may minimize product inhibition and, in turn, boost carbon utilization.

Cluster 2 (p-value 1.11e-15; yellow nodes) is enriched in proteins related to cellular transport functions, including establishment of localization (GO:0051234) and ion transport (GO:0006811). Notably, TBDRs make up two-thirds of the cluster, while the remaining proteins include three unknown proteins (E7_MBN_00645, E7_MBN_02552 and E7_MBN_4031). Two TBDRs (E7_MBN_01610 and E7_MBN_01622) were found to be localized in a polysaccharide utilization locus (PUL) that target xyloglucan and pectin, reflecting their critical role in the assimilation of plant cell wall-derived sugars. TBDRs are present in all Gram-negative bacteria, serving as a transport system for nutrient acquisition in resource-limited environments (Tang et al. 2012). In *T. turnerae*, a recent study by Naka and Haygood showed that TBDRs are essential for iron and carbohydrate uptake, with evidence showing several tonB genes influence the growth of bacteria in cellulose (Naka and Haygood 2023). Meanwhile, unknown proteins in this cluster could also be implicated in extracellular transport. For example, E7_MBN_04031 features a PKD domain, which was suggested to mediate the transport of carbohydrate oligomers from CAZymes to TBDRs for subsequent assimilation (Kabisch et al. 2014). These putative transport proteins were predicted to co-occur across different bacterial genomes, particularly those that thrive in a broad range of aquatic ecosystems, suggesting that these may be key proteins for adaptation in these specific environments.

Interestingly, several CAZymes grouped together to form Cluster 3 (p-value = 7.26e-06; green nodes). This cluster includes pectate lyase (E7_MBN_01628), AA10 LPMO (E7_MBN_01171), two CBM-containing proteins (E7_MBN_00260 and E7MBN_03841), and an unknown protein (E7_MBN_00021). Although no significant functional enrichment was detected for this cluster, save for the presence of CBD_II domain in some proteins, we can infer that these proteins may potentially serve a role in degrading PCWPs. It is interesting to note that CAZymes within this cluster consist of non-hydrolytic enzymes, suggesting that these proteins act together on carbohydrates through a different mechanism. This cluster is also closely associated with the first cluster, indicating possible cooperation between the two enzyme systems in plant cell wall utilization.

The unknown protein (E7_MBN_00021) in Cluster 3 appears to interact with AA10 LPMO and GH5 cellodextrinase. This protein is homologous to a lipoprotein in T7901 (UniProt: C5BRG0) and aligns with the cytochrome c domain-containing protein cluster UniRef50_A0A1Y0G3J5 from *Cellvibrio sp. PSBB006*. Electron donors, in the form of proteins like cellobiose dehydrogenase, or small molecule reductants such as ascorbic acid, are essential for activating the catalytic copper in LPMOs (Kracher et al. 2016). Structural analysis of the AA10 LPMO from *T. turnerae* T7901 revealed a second metal-binding site, suggesting a possible role for a protein-based activator in the LPMO’s electron transfer system (Fowler et al. 2019). Given such sparse functional information on E7_MBN_00021, it is difficult to ascribe a role for this unknown protein. However, given that we only detect this in secretomes where AA10 LPMO is present and with their association in the PPI network, these can be clues to the possibility that this unknown protein could be a redox partner for AA10 LPMO.

Cluster 4 (p-value = 2.08e-04; blue nodes) consists of an isomerase (E7_MBN_02352), a superoxide dismutase (SOD, E7_MBN_03304), and a ferritin-like protein (E7_MBN_03876). Unlike other clusters, Cluster 3 exhibited completely linear connectivity with no overlapping interactions and lacked functional enrichment. Isomerases in this cluster may play a role in cellular metabolism, facilitating the entry of hydrolytic products from PCWPs into various metabolic pathways, as suggested by the interaction with Cluster 1. The inclusion of SOD, an enzyme typically involved in oxidative stress response, within this cluster is intriguing and raises the possibility of its protective role during PCWP breakdown, wherein oxidative processes may occur.

**Fig. 4:**
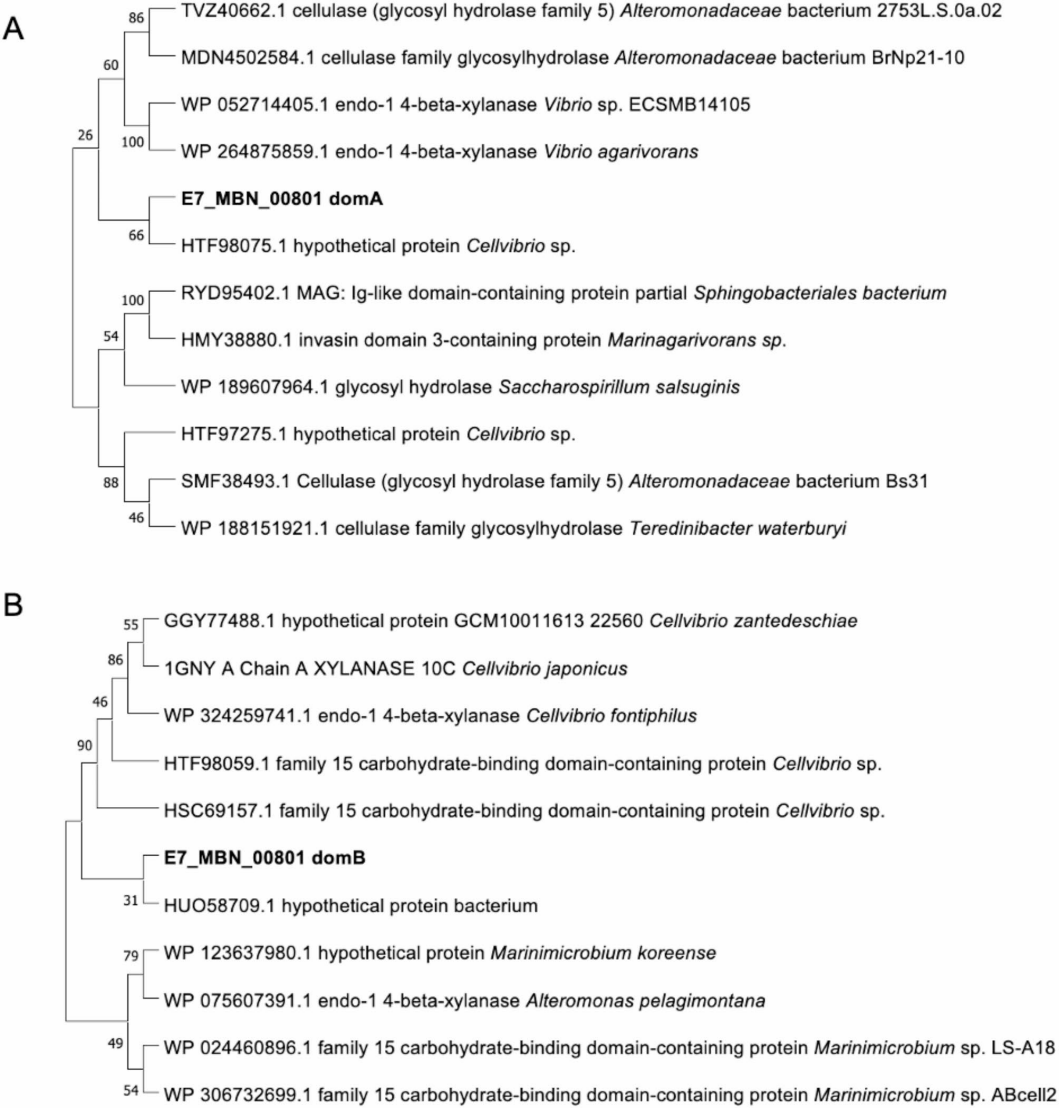
Phylogenetic trees for E7_MBN_00801 domains (**A**) domA and (**B**) domB constructed using the neighbor-joining tree method. Bootstrap values were obtained after 1000 iterations

Intercluster relationships, represented as dashed lines, in the PPI network can be observed, mediated by specific proteins in Cluster (1) Curiously, the unknown protein E7_MBN_00801 appears to link Cluster 1 to Cluster 2, interacting with hydrolytic enzymes (E7_MBN_00820 and E7_MBN_00821) in Cluster 1 and a tonB-dependent receptor protein (E7_MBN_01278) in Cluster (2) While these associations suggest its potential role in carbohydrate degradation and transport, bioinformatic functional analysis of E7_MBN_00801 provided conflicting results. E7_MBN_00801 is annotated as hypothetical GH8 family cellulase protein via Prokka, while domain analysis by InterPro only predicted the presence of galactose-binding domain-like motifs and dbCAN3 did not find any known CAZyme domain. BLASTP analysis showed that E7_MBN_00801 exhibits low sequence similarity with other bacterial CAZymes, with the top hit being a CBM15 domain-containing protein form *Marinibacterium* sp. LS-A18 (Genbank Accession: WP_024460896.1, 32.24% identity, 97% coverage). Structural investigation of the protein revealed two distinct domains, referred to as domA and domB, each adopting a β-jelly roll fold characteristic of many CBM families (Figure S2) (Boraston et al. 2004). To infer the putative function of the protein, phylogenetic trees were constructed for each domain (Fig. 4). The two E7_MBN_00801 domains seem to be distantly related to proteins that act on hemicellulose, with domA being associated with GHs and domB linked to xylan-binding proteins. Particularly, the phylogenetic tree for domB includes a characterized CBM15 xylan-binding module from *Cellvibrio japonicus* (PDB: 1GNY) (Szabó et al. 2001), indicating a potential functional relationship. Given that this protein is exclusively secreted in the presence of xylan, these results point to E7_MBN_00801 as a protein that can potentially interact with xylan substrates.

### Residual biomass utilization of *Teredinibacter turnerae*

Finally, to assess whether E7MBN can utilize residual biomass, we cultured the bacterium in SBM supplemented with rice hull, referred to as E7R. Both CMCase and xylanase activities were detected in the E7R media (data not shown), similar to our observations in other growth conditions. The E7R secretome comprises 14 proteins (Table S1), and despite having fewer proteins than those from model substrates, almost half of these (6 proteins) are identified as CAZymes. Consistent with our previous results, CAZymes identified in E7R are also common to E7C, except for an endoxylanase/acetylxylan esterase (E7_MBN_00238) which was only identified in E7R. This multicatalytic enzyme features both CE6 and GH10 domains, similar to E7_MBN_00081 (69.1% identity, 59% coverage). The AA10 LPMO appears to be exclusively expressed between E7R and E7C, along with the hypothetical protein E7_MBN_00021, which has been predicted to interact with LPMO in the PPI network. Furthermore, the proteomic profile of the rice hull-grown secretome highly resembles that of secretome grown in cellulose, similar to what was observed for the secretome supplemented with sucrose.

## Discussion

In this study, we employed a shotgun proteomics approach to investigate the plant cell wall degradation system in *T. turnerae* and identify the proteins secreted during growth in minimal media and major PCWPs (i.e., cellulose, xylan, and pectin). We also report an initial assessment of *T. turnerae*’s ability to degrade rice hull biomass, providing a glimpse into its potential for lignocellulose degradation and industrial applications. Our results show that *T. turnerae* E7MBN strain exhibits minimal secretion when grown in different carbon sources. Proteomic profiles showed minimal variation between minimal media and PCWP growth conditions, with most CAZymes identified across multiple substrates. Enzymatic complexity, however, varied depending on the substrate. Cellulose-grown secretomes exhibited the highest complexity, containing nearly all the CAZymes detected. In contrast, growth on other carbon sources resulted in fewer CAZymes, with only a single CAZyme identified in the xylan-grown secretome. We also highlight the consistent involvement of multicatalytic CAZymes in the secretome, possibly serving a central role in PCWP deconstruction. Finally, PPI network analysis provided an in-depth evaluation of the functional relationships within the secretome, uncovering previously uncharacterized proteins with potential roles in lignocellulose degradation.

Our results show loose correlation between CAZyme secretion and the carbon source. Secretomes grown in sucrose, cellulose, and pectin displayed activity against multiple substrates and contained CAZymes with diverse catalytic domains. These include representatives of five GH families (GH5, 6, 10, 11, 51), two CE families (CE1 and CE6), and two PL families (PL1 and PL11). Each of these families target bonds found within cellulose, hemicellulose, or pectin, enabling the breakdown of plant cell walls. CBM families (CBM2, 4, 5, 10, 35, 60), which bind primarily to cellulose or hemicellulose components, were also identified across the secretomes. The broad catalytic activities observed for proteins produced by *T. turnerae* E7MBN under these growth conditions align with previous studies, which also report a wide range of CAZyme activities when the bacterium is grown in pure cellulose and within the gills of shipworms (O’Connor et al. 2014; Sabbadin et al. 2018; Yang et al. 2009). Although cellulose-grown secretome induced the most number of CAZymes, the functional profiles across other growth conditions, except for xylan, are arguably similar, suggesting that CAZyme expression in *T. turnerae* is largely constitutive under various conditions, including cellulose, sucrose, and pectin. This may reflect a mechanism similar to other bacteria, such as *Clostridium cellulolyticum*,* Corallococcus silvisoli*, and *Caldicellulosiruptor*, which have been reported to produce high levels of lignocelullolytic enzymes in the presence of mono- or oligosaccharides or across multiple polysaccharides (Lochner et al. 2011; Xu et al. 2013; Zhou et al. 2024). While transcriptomic evidence is necessary to fully understand the regulation of CAZyme expression in *T. turnerae*, our current knowledge of its ecological niche suggests that this may be a result of its restricted environment in the shipworm gills. Unlike other symbiotic bacteria that resides in the host’s gut, such as those in termites (Ali et al. 2019; Salgado et al. 2024), the *T. turnerae* bacterium is found in specialized cells (“bacteriocytes”) located in the gills, far from the cecum where digestion primarily takes place (O’Connor et al. 2014; Sabbadin et al. 2018). Therefore, in an environment where it has no direct contact with substrates, *T. turnerae* may have adapted this strategy to readily supply enzymes to its host without relying on specific polysaccharides for induction.

On the other hand, the secretion of a single CAZyme in the presence of xylan could indicate a more selective or limited response to hemicellulosic substrates compared to other PCWPs. The fact that only one xylanase/acetylxylan esterase (E7_MBN_00081) was found in xylan is quite intriguing given the presence of multiple hemicellulases in other conditions. Furthermore, this same enzyme was also the only CAZyme identified in all carbon sources, which may point to its central role in *T. turnerae*’s enzymatic repertoire and its potential involvement in a more specialized response to hemicellulose. It can also be argued that the limited response of *T. turnerae* E7MBN to xylan could indicate a potentially different regulatory mechanism for xylan degradation. Transcriptomic analysis of *Cellvibrio japonicus* revealed distinct regulatory responses to different lignocellulosic substrates (Blake et al. 2018). While cellulose induced the expression of a diverse array of CAZymes that target various polysaccharides, only xylan-specific CAZymes were upregulated when the bacterium was grown in oat-spelt xylan (Blake et al. 2018). It would be interesting to know whether *T. turnerae* operates through a similar mechanism, and if this response is specific to xylan or also extends to other polysaccharides.

A redundancy of hemicellulases was observed across all secretomes. Interestingly, these enzymes are all multicatalytic and mostly feature a combination of GH and CE domains. Multicatalytic CAZymes are commonly found in plant biomass-degrading microorganisms, such as *Caldicellulosiruptor* and *Bacteriodites*, where several novel enzymes have been identified and biochemically characterized (Brunecky et al. 2017; Chu et al. 2019; Conway et al. 2018; Kmezik et al. 2020, 2021; Krska et al. 2021). In *T. turnerae*, a high prevalence of multicatalytic CAZymes has also been reported (Brito et al. 2018; Yang et al. 2009). However, only a single multifunctional cellulase, CelAB, has been characterized to date (Ekborg et al. 2007). While multicatalytic CAZymes are relatively understudied compared to other enzymes, co-localization of multiple catalytic domains within one polypeptide chain has been shown to enhance synergy in most studies, resulting in improved degradation efficiency (Brunecky et al. 2017; Chu et al. 2019; Kmezik et al. 2020). Here, we propose that the consistent presence of these enzymes in the secretome serves as a strategy employed by *T. turnerae* to ensure broad substrate specificity and catalytic efficiency, all while secreting fewer enzymes. This aligns with our initial observation of minimal enzyme secretion, suggesting that the bacterium relies on a core set of enzymes to degrade various plant cell wall components. Given that *T. turnerae* is an aerobic bacterium, its cellulolytic machinery relies on secreted free enzymes rather than cellulosomes, which are multiprotein complexes exclusively found in anaerobic bacteria (Artzi et al. 2017; Zhivin-Nissan et al. 2019; Phitsuwan et al. 2019). Thus, in the absence of cellulosomes, *T. turnerae* produces free enzymes that are both multidomain and multicatalytic. This strategy offers the advantages of cellulosomes, such as localized enzymatic activity and substrate binding, while also benefiting from reduced energy cost and enhanced mobility typical of free enzymes. Although it is uncertain whether these proteins exhibit diverse substrate specificities or operate through distinct mechanisms, the redundancy of multicatalytic hemicellulases could indicate their key function in *T. turnerae*’s proficiency for breaking down terrestrial polysaccharides. However, this also raises interesting questions on the factors that regulate the expression of multicatalytic CAZymes in *T. turnerae* and whether they are selectively or constitutively expressed and secreted under different culture conditions.

The secretion of multifunctional CAZymes as free enzymes can potentially complement outer membrane vesicles (OMVs) that have been recently shown to facilitate the delivery of *T. turnerae* enzymes into the environment (Gasser et al., 2024). It has been reported that *T. turnerae* secretes OMVs that contain a broad range of CAZymes which retain significant cellulolytic activity (Gasser et al. 2024). This presents an alternative pathway for protein transport, allowing localization and concentrated release of proteins for enhanced degradation. In comparison with our results, only two multicatalytic CAZymes were identified in the MV proteome, including the glycoside hydrolase family 11 and family 5 domain protein (TERTU_0428), homologous to E7_MBN_00820, and the multifunctional cellulase, CelA1 (TERTU_2895), which contains GH5 and GH6 domains. This is relatively few compared to the number of identified CAZymes in the MV proteome, leading us to speculate that *T. turnerae* may employ a complementary secretion mechanism. In this context, while OMVs package and deliver multiple enzymes collectively, it is possible that most multicatalytic CAZymes are secreted as free enzymes. This confers functional versatility, coordinated regulation, and substrate channeling or better spatial organization, while also allowing the bacteria to transport proteins efficiently. Altogether, both strategies highlight the significance of coordinated activity in degrading complex substrates and suggest a dual system by which *T. turnerae* deliver and utilize its enzymes during PCWP deconstruction. In future studies, it would be interesting to determine the interplay between these two secretion mechanisms, whether specific CAZymes are preferentially secreted in OMVs or as free proteins, and how these pathways are regulated in response to different substrates.

Other non-CAZyme proteins that may participate in PCWP utilization in *T. turnerae* were also identified. Among these, tonB-dependent receptors (TBDRs) emerged as common components of the secretome, representing the majority of transport proteins during breakdown of plant cell walls. These proteins mediate the energy-dependent transport of various substrates, such as siderophores, oligosaccharides, and other nutrients, across the bacterial outer membrane. It has been reported that genes encoding for TBDRs are conserved among several lignocellulolytic marine bacteria, such as *Xanthomonas campestris pv. campestris*,* Caulobacter crescentus*, and *Saccharophagus degradans*, suggesting their critical role in the acquisition of plant-derived carbohydrates (Blanvillain et al. 2007). *C. crescentus* was shown to use the TBDR-mediated transport in scavenging glycans, such as cellobiose and other oligosaccharides, derived from degradation of cellulose (Presley et al. 2014). TBDRs were also demonstrated to participate in lignocellulose utilization in *Sphingobium sp*. strain SYK-6, facilitating uptake of lignin-derived aromatic compounds (Fujita et al. 2019). In *T. turnerae*, knockout experiments revealed that two *TonB* genes are essential for the bacteria’s growth in iron-limiting conditions and with cellulose as its sole carbon source, highlighting important role of TBDRs in iron acquisition and carbon sequestration (Naka and Haygood 2023).

Proteins with peculiar domain architecture, such as the CBM-containing redox enzymes, and previously uncharacterized proteins, including the putative hemicellulose-acting protein, E7_MBN_00801, were also reported in this study. The complex modular domain architecture of *T. turnerae* redox proteins may be unique to this bacterium, and elucidating the role and interplay of the different domains makes it an exciting target for further biochemical characterization. PPI network analysis provided clues to the possible functions of several uncharacterized proteins, and their predicted functional associations with other secreted proteins suggest a supporting role in carbohydrate utilization, likely contributing to *T. turnerae*’s overall enzymatic efficiency.

## Conclusion

Enzyme systems employed by plant biomass-degrading microorganisms possess great potential for sustainable production of fuels and chemicals. With the emerging relevance of bacterial CAZymes in biomass conversion strategies, secretome analysis of novel cellulolytic bacteria could be beneficial to the discovery of industrially relevant enzymes and understanding of unique degradative mechanisms. This study provides protein-level evidence of *T. turnerae* E7MBN’s versatile capability for plant biomass degradation. Our findings demonstrate that *T. turnerae* E7MBN employs a minimal yet effective set of CAZymes, notably multicatalytic enzymes, enabling a highly coordinated enzyme system capable of targeting a wide variety of substrates. These proteins may be crucial to developing sustainable, low-complexity enzyme mixtures that can be used for various industrial and biotechnological applications. Limited variation in enzyme secretion across different substrates was also observed, with *T. turnerae* only demonstrating a more specific response to xylan. This could indicate distinct regulatory mechanisms for hemicellulose degradation and warrants further investigation. Correspondingly, the *T. turnerae* secretome may also be used as model in designing minimal enzyme mixtures, which include few but efficient enzymatic components, allowing for a more optimized and cost-effective bioconversion strategy. Furthermore, the identification of non-AA redox enzymes, TBDRs and other associated proteins suggests a larger, integrated protein system in *T. turnerae*, offering an interesting platform for the discovery of unique proteins involved in carbohydrate utilization. Lastly, initial studies on growth in waste biomass highlight *T. turnerae*’s potential for complete plant cell wall degradation and present exciting opportunities for developing efficient biocatalytic processes to produce value-added products from agricultural waste feedstocks.

## Electronic supplementary material

Below is the link to the electronic supplementary material.

Supplementary Material 1

## Data Availability

The authors confirm that the data supporting this study’s findings are available within the article and in the supplementary information files. The datasets used and analyzed in this study are available as partial submissions in MassIVE and ProteomeXchange repositories with the identifiers MSV000097320/PXD061820 (DOI: https://doi.org/doi:10.25345/C5319SF5Z).

## Abbreviations

PCWP: Plant cell wall-associated polysaccharides
CAZymes: Carbohydrate-active enzymes
GH: Glycoside hydrolase
PL: Polysaccharide lyase
AA: Enzyme with auxiliary activity
LPMO: Lytic polysaccharide monooxygenase
CBM: Carbohydrate-binding module
TBDR: TonB-dependent receptor
PPI: Protein-protein interaction
