# Supplementary material for: Teredinibacter turnerae secretome highlights key enzymes for plant cell wall degradation
Source: Bioresour Bioprocess. 2025 May 6;12(1):42. doi: 10.1186/s40643-025-00876-7 (PMC12055684; doi:10.1186/s40643-025-00876-7)
Supplement: Supplementary file 1 — Supplementary Material 1 [file 40643_2025_876_MOESM1_ESM.docx]

**Supporting Information**

**Figure S1: SDS-PAGE profile of culture supernatants grown in sucrose (E7S), cellulose (E7C), xylan (E7X), and pectin (E7P).** Proteins were loaded at 5 µg total protein concentration in 12% polyacrylamide gel. M stands for Precision Tricolor Broad Range Prestained Protein Ladder (Vivantis); protein markers are in kDa.


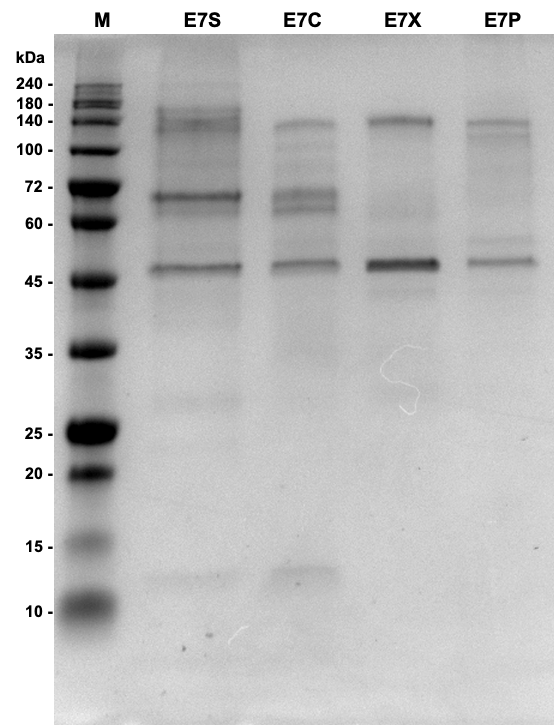


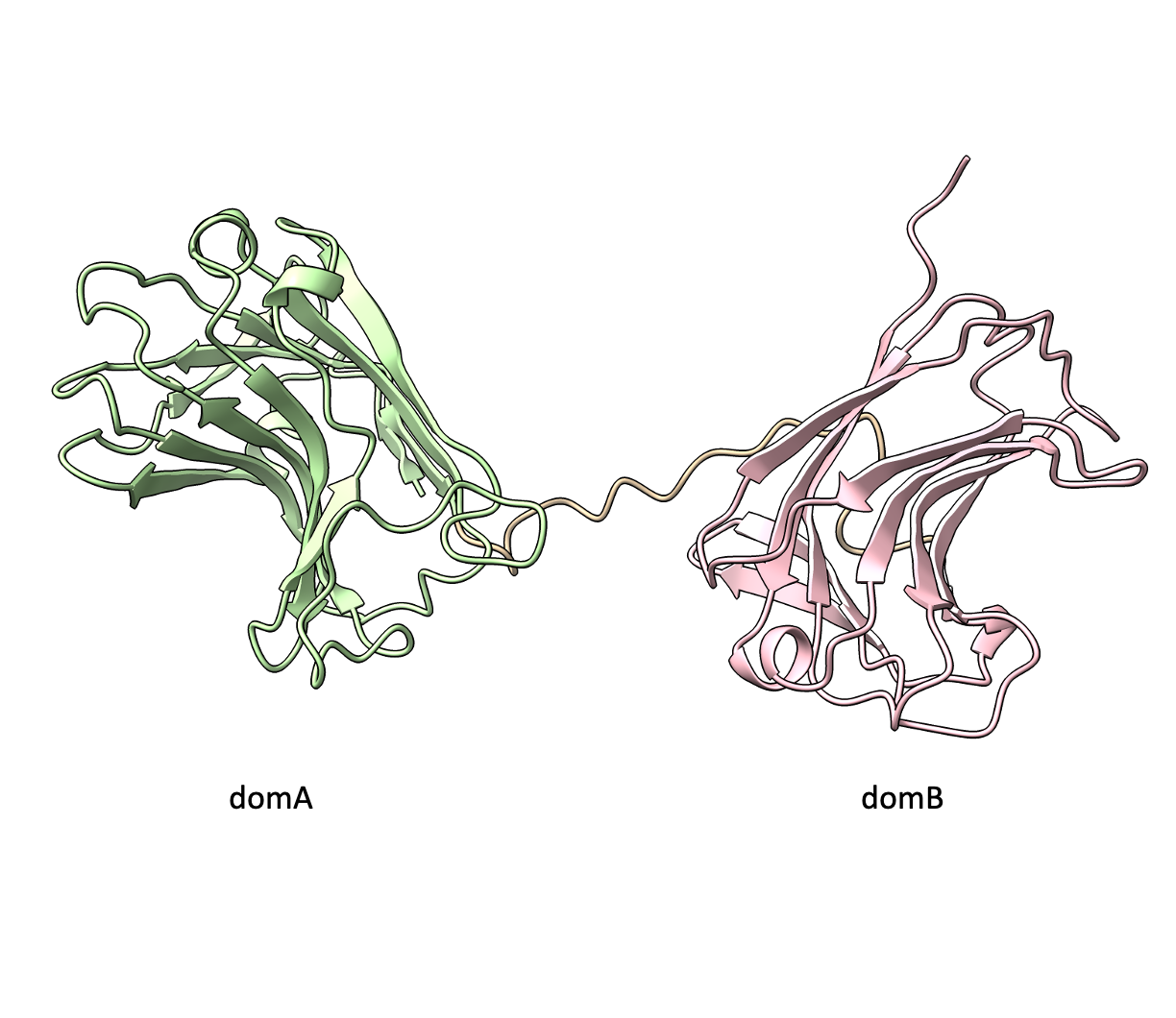
**Figure S2. E7_MBN_00801 has two distinct domains, domA and domB.** The putative structure of E7_MBN_00801 was predicted using AlphaFold 3 (Abramson et al., 2024).

**Table S1. Proteins identified in the culture supernatant of *T. turnerae* E7MBN grown on rice hull biomass by LC-MS/MS.** T7901 homologs were identified using the UniProt database, and signal peptides were predicted via SignalPv6.0. A non-classical secretion mechanism was assigned to proteins lacking signal peptides and scoring above 0.5, as predicted by the Secretome 2.0 server.

| Accession code | Annotation | UniProt accession | Mass (Da) | Signal peptide | Predicted secretion  mechanism | Presence in  other substrates |
| --- | --- | --- | --- | --- | --- | --- |
| E7_MBN_00021 | hypothetical protein | C5BRG0 | 70405 | N | Non-classical | C |
| E7_MBN_00238 | Ca-dependent carbohydrate-binding module xylan-binding | C5BRL9 | 115377 | Y | Classical | - |
| E7_MBN_00239 | endoxylanase/acetylxylan esterase | C5BQU7 | 126570 | Y | Classical | C, P |
| E7_MBN_00260 | carbohydrate binding protein | C5BSV8 | 198086 | Y | Classical | S, C |
| E7_MBN_00497 | Unknown protein | K7FWY9 | 22618 | N | - | S, X, P |
| E7_MBN_00820 | glucanase/endoxylanase | C5BMU2 | 97022 | Y | Classical | C, R |
| E7_MBN_01171 | Lytic polysaccharide monooxygenase | C5BKQ9 | 35628 | Y | Classical | S, C |
| E7_MBN_01277 | TonB-dependent Receptor Plug Domain | C5BKF0 | 125860 | Y | Classical | S, C, P |
| E7_MBN_02842 | TonB dependent receptor | C5BN15 | 111955 | Y | Classical | S, C, X, P |
| E7_MBN_03193 | Belongs to the glycosyl hydrolase family 6 | C5BNB1 | 40736 | N | Non-classical | C, P |
| E7_MBN_03304 | Iron/manganese superoxide dismutases, alpha-hairpin domain | C5BP07 | 21574 | N | Non-classical | S, C, X, P |
| E7_MBN_03305 | TCP-1/cpn60 chaperonin family | C5BP08 | 57033 | N | - | - |
| E7_MBN_04122 | Repeats in polycystic kidney disease 1 (PKD1) and other proteins | C5BHW7 | 129837 | Y | Classical | - |
| E7_MBN_04207 | Glutamine synthetase, catalytic domain | C5BJ19 | 52058 | N | - | P |
